# Study on Crack Resistance Mechanism of Helical Carbon Nanotubes in Nanocomposites

**DOI:** 10.3390/nano15020119

**Published:** 2025-01-15

**Authors:** Zhiwu Bie, Xuefeng Liu, Yajie Deng, Xian Shi, Xiaoqiao He

**Affiliations:** 1 Department of Architecture and Civil Engineering, City University of Hong Kong, Tat Chee Avenue, Hong Kong; zwbie2-c@my.cityu.edu.hk (Z.B.); bcxqhe@cityu.edu.hk (X.H.); 2 College of Hydraulic and Environmental Engineering, China Three Gorges University, Yichang 443002, China; xfliu6-c@my.cityu.edu.hk; 3 School of Aerospace Engineering and Applied Mechanics, Tongji University, Shanghai 200092, China; 4 School of Civil Engineering, Suzhou University of Science and Technology, Suzhou 215009, China; shixian@usts.edu.cn

**Keywords:** helical carbon nanotubes, nanocomposites, crack resistance, molecular dynamics

## Abstract

Helical carbon nanotubes (HCNTs) with different geometrical properties were constructed and incorporated into nanocomposites for the investigation of the anti-crack mechanism. The interfacial mechanical properties of the nanocomposites reinforced with straight carbon nanotubes and various types of HCNTs were investigated through the pullout of HCNTs in the crack propagation using molecular dynamics (MD). The results show that the pullout force of HCNTs is much higher than that of CNTs because the physical interlock between HCNTs and matrices is much stronger than the van der Waals (vdW) interactions between CNTs and matrices. Remarkably, HCNTs with a large pitch length can not only effectively prevent the initiation of breakages but also hinder the growth of cracks, while HCNTs with a small diameter and tube radius cannot even effectively prevent the initiation of cracks, which is similar to straight CNTs. Moreover, the shear resistance of HCNTs increases with the increase in the helix angle, which remains at a high level when the helix angle reaches the critical value. However, HCNTs with a small helix angle and large diameter can carry out more polymer chains, while snake-like HCNTs and HCNTs with a small diameter and helix angle can hardly carry out any polymer chain during the pullout process and show similar interfacial properties to the straight CNTs.

## 1. Introduction

Carbon-based materials (such as carbon fibers, carbon nanotubes, graphene, diamond nanothread, fullerene, etc.) have been widely applied as reinforcements in the polymer matrix of nanocomposites because they possess remarkable mechanical properties, such as superior ductility, excellent energy absorbing ability, and reversibility [1,2,3,4,5,6,7,8,9,10,11]. The effect of carbon-based materials on the improvement of mechanical behaviors of nanocomposites has been investigated comprehensively [12,13,14,15]. Tang et al. [16] experimentally found that the reinforcement of multi-walled carbon nanotubes (MWCNTs) in nanocomposites was attributed to three anti-fracture modes, and the pullout behaviors of MWCNTs from the matrix was the common pattern in all three fracture modes. The increases of up to 59% in fracture toughness by adding MWCNTs were also found to be attributed to the three anti-fracture modes. Moreover, Quan et al. [17] found that the incorporation of MWCNTs could moderately increase the fracture energy of mode-I and mode-II cracks, which was attributed to crack bridging (nanotube pullout), nanotube breaking, and crack deflection.

To investigate the effect of the various parameters of carbon-based materials on the fracture mechanical properties of nanocomposites, the pullout test was widely used to study the interfacial properties between reinforcing fillers and the matrix. Duan et al. [13] found that the interfacial shear strength was affected by the diameter and length of CNTs. Zheng et al. [18] investigated the effects of the radius and chirality of CNTs on the interaction between CNTs and polymers. The results showed that the interfacial strength between CNTs and the polymer matrix was highly dependent on chirality. Liu [19] demonstrated that the interfacial mechanical properties of nanocomposites were highly improved by introducing a wrinkled graphene sheet. In addition, the nanocomposites with graphene were constructed by putting polymer chains on the surface of graphene, and the force-separation behavior between graphene and the polymer matrix under opening and sliding modes was investigated by Awasthi et al. [20]. The results showed that interfacial failure occurred within the polymer region, and the obtained interfacial mechanical properties can be used to investigate the properties of nanocomposites under continuum assumptions. Similarly, Li et al. [21] also simplified the CNT/polymer nanocomposites to the use of graphene–polymers, and the loading transfer between graphene and polymer was investigated. However, the structure of graphene–polymer composites was different from that of CNT/polymer composites, where the polymer chains were entangled and wrapped around CNT. Furthermore, cohesive zone traction–displacement laws were developed based on the mechanical properties obtained from the MD simulations used to study the effect of the interface on the macroscale effective elastic properties through continuum-level models.

The defects in the CNTs and graphene can also affect the mechanical behaviors of nanocomposites [4,22,23,24,25,26]. For example, Stone–Wales (SW) defects could improve transverse Young’s modulus and the transverse/longitudinal shear moduli of the composites due to stronger interfacial adhesion between the defected CNTs and matrix [4]. Peng et al. [24] found that vacancy defects degraded the interfacial strength, and the main factor was the number of missing atoms instead of the type of vacancy defects. Moreover, longitudinal Young’s modulus for the nanocomposite increased as the number of SW defects decreased [22]. The functionalization of CNTs and graphene was also believed to improve the load transfer between the polymer matrix and nanofillers to obtain higher fracture toughness by increasing the interlock strength and contact area between the nanofillers and matrix [24,27,28,29,30,31].

The above studies show that the improvement in the mechanical properties of nanocomposites is mainly attributed to the interfacial properties between the matrix and nanofillers. Since the interfacial property is weak due to the ineffective vdW interaction without efficient interlock between straight CNTs/the graphene and polymer matrix, various methods, such as the construction of the wrinkled surface [19], functionalization [27,29,31] and introduction of defects into straight CNTs [4,23,24], have been proposed to improve the interfacial strength of nanocomposites. However, these methods are either complex or inefficient to use in practical applications. Fortunately, the HCNTs with a special 3D spiral structure can be mechanically interlocked with the matrix and cannot be pulled out from the matrix easily, which improves the efficiency of load transfer between the nanofillers and the matrix [32,33,34,35,36,37,38]. Furthermore, HCNTs possess superior elastic properties and energy-absorbing capacity during the deformation process [39,40,41,42], and the HCNTs were found to be more excellent in hardening the matrix than CNTs by three times [32]. Through the experiments, Li et al. [34] studied the reinforcement mechanism of HCNT fillers. It was shown that HCNTs were broken instead of being pulled out from the matrix under the external load, which can be explained by the superior efficiency of the load transfer to the HCNTs from the matrix. Also, the experimental results showed that HCNTs were dispersed well in the matrix. Moreover, the effect of the diameter and functionalization of HCNTs, the temperature, and the number of polymer chains on the interfacial mechanical properties of nanocomposites was also investigated [35]. The results showed that the functionalization and diameter of HCNTs had an obvious effect on the mechanical properties of nanocomposites.

Although the nanocomposites reinforced by the HCNTs have been investigated theoretically and experimentally, only the HCNTs with a small pitch length where the inter-tube gap of the HCNTs is nearly equal to vdW interaction distance have been studied in the nanocomposites. In addition, the pitch length and geometric properties of spring-like structures can significantly affect the reinforcement and load transfer in nanocomposites [43]. Therefore, the interfacial mechanical properties of nanocomposites reinforced by HCNTs with different pitch lengths, helix angles, and diameters were systemically studied in this work. Moreover, the effect of geometrical properties of HCNTs on their ability to hinder the initiation and growth of cracks is comprehensively studied, and the obtained results can help to design nanocomposites with excellent mechanical properties.

## 2. Model Construction in MD Simulation

Before the construction of nanocomposites, the HCNTs with different geometrical properties are constructed using the method outlined in Ref. [44]. We used the symbol (n75, n55, s, os = m) to denote the spiral structure of HCNTs, where n75 is the number of hexagons between the heptagon and pentagon along the peripheral direction of CNT segments at the corner of toroidal CNTs (TCNTs), n55 is the number of hexagons between two pentagons, s is the number of hexagons between two pentagons along the axial direction of CNT segments and os is the number of hexagons between two pentagons located on the outer-rim of TCNTs along the latitudinal direction after rotation, which can be seen as the distortion degree. It is noted that a snack-like HCNT with an irregular shape is constructed by randomly linking the different curved CNT segments, which cannot be denoted by the parameters mentioned above.

In order to identify the HCNTs clearly, two more indices were used, i.e., (n75, n55, s, os = m)-*p-D*, where *p* is the pitch length, and *D* is the effective diameter of HCNTs which can be calculated as follows:

(1) $$D=\frac{2}{N}\sum_{i=1}^{N}\sqrt{\left({\left(x_{i}-x_{\mathrm{center}}\right)}^{2}+{\left(y_{i}-y_{\mathrm{center}}\right)}^{2}\right)}$$

where *x*_center_ and *y*_center_ are the center positions in the *x-y* plane of HCNTs containing *N* atoms. *x_i_* and *y_i_* refer to the position of atom *i* in the *x-y* plane, and the helix angle *α* of the HCNTs is calculated by the following:

(2) $$\alpha =\arctan(\frac{p}{\pi D})$$

**Figure 1:**
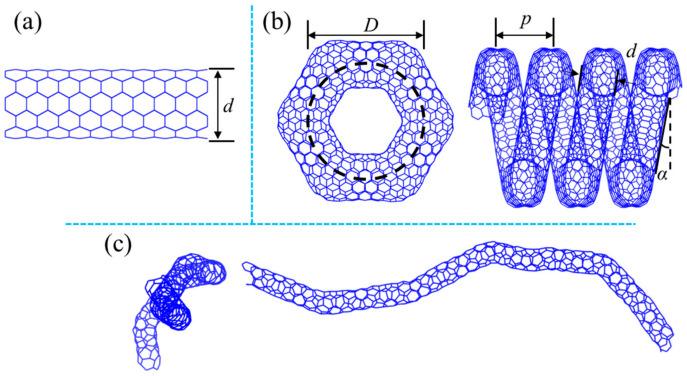
(**a**) Straight CNTs with diameter *d*; (**b**) diagram of HCNTs with the demonstration of geometrical parameters; and (**c**) special snake-like HCNTs.

The detailed geometrical properties are shown in Table 1 and Figure 1. It is noted that the snake-like HCNT is constructed based on straight CNTs with a small tube diameter and high helix angle, as shown in Figure 1c.

**Figure 2:**
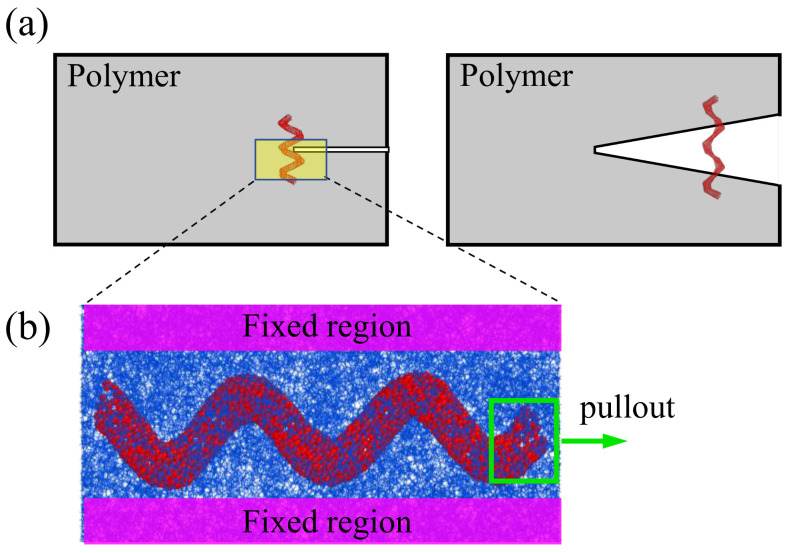
(**a**) The diagram of nanocomposites reinforced by HCNTs with an initial crack and the diagram of HCNTs that hinder the propagation of crack; (**b**) simplified atomic models of nanocomposites with HCNTs.

From the microscale, the nanofillers were randomly distributed in the polymers. However, the formation of fractures always exists on the interface of the nanofillers and the polymer matrix, and the reinforcement of the nanofillers is attributed to the pullout of nanofillers, which is commonly used to investigate the crack resistance of nanofillers. Hence, the nanocomposites with the HCNTs were constructed to investigate the interfacial effect of the HCNTs with various structural parameters, as shown in Figure 2. It can be seen that when the fracture occurred in the nanocomposites, the nanofillers helped to hinder the initiation and growth of cracks in the nanocomposites. The strong physical interlock between HCNTs and the polymer matrix, induced by the 3D helical structure, can hinder not only the initiation of fracture but also the growth of cracks, as shown in Figure 2a.

Polyethylene (PE) was selected as the polymer matrix since it has been widely used to investigate the mechanical properties of nanocomposites due to its simple structure. Herein, each PE chain contained 20 repeating units of CH_2_. One HCNT was first placed in the middle of the simulation cell, and then PE chains were packed into the simulation cells, as shown in Figure 2b.

In order to obtain a stable nanocomposite structure, all nanocomposite models were relaxed under the NVT ensemble at the temperature of 300 K for 200 ps, followed by relaxation under the NPT ensemble at the temperature of 300 K and pressure of 1 atm for another 200 ps. The structures were then relaxed under the NVT ensemble for another 100 ps to obtain the final equilibrium structure. A time step of 0.5 fs was chosen for all relaxation procedures [45].

## 3. Simulation Procedure

As shown in Figure 2, a vacuum with a thickness of 200 Å was added in the axial direction of the box to allow the pullout of nanofillers from the PE matrix. The boundary condition along the axial direction was changed to non-periodic, while periodic boundary conditions remained in other directions. A constant velocity of 0.00015 Å/fs [35] was applied at one end of the nanofillers under the NVT ensemble. During the pullout process, the loading part of the nanofillers was set as a rigid body while the other part was flexible, and the PE atoms at the lateral edges were fixed with a certain thickness according to the size of the box, as shown in Figure 2.

The pullout test was utilized to investigate the interfacial interaction between the polymer matrix and nanofillers, which is the main factor affecting the reinforcement of nanocomposites. All molecular simulations were implemented in LAMMPS 29Sep2021. The consistent valence forcefield (CVFF) was used to simulate the interactions between atoms, which has been successfully used to study carbon-based nanocomposites [20,21]. The CVFF potential can be expressed as follows:

(3) $$E_{total}=\sum_{b}K_{b}{\left(b-b_{0}\right)}^{2}+\sum_{\theta }K_{\theta }{\left(b-b_{\theta }\right)}^{2}+\sum_{\varphi }K_{\varphi }\left[1+scos(n\varphi )\right]\;+\sum_{\chi }K_{\chi }\;[1-cos(2\chi )]+\sum_{nonbond}\left\{\varepsilon_{ij}\left[{\left(\frac{r_{ij}^{0}}{r_{ij}}\right)}^{12}-{\left(\frac{r_{ij}^{0}}{r_{ij}}\right)}^{6}\right]+\frac{q_{i}q_{j}}{\varepsilon_{0}r_{ij}}\right\}$$

The total energy *E_total_* consists of the bonded energy terms and the non-bonded energy terms. The first four terms are bonded interactions, including the covalent bond energy, the bond angle bending energy, the torsion angle twisting energy, and the out-of-plane deformation energy. The last term represents the non-bonded interactions, which include an LJ-12-6 function for the van der Waals (vdW) term and a Coulombic function for the electrostatic interactions. The cut-off distance was chosen as 12 Å [46].

## 4. Results and Discussions

To investigate the effect of the geometries of HCNTs on the interfacial strength between the nanofillers and matrix, the pullout force was obtained by the sum of forces along the pullout direction from the PE matrix. Then, the pullout force applied per atom was calculated by dividing the pullout force by the atoms of nanofillers since the number of atoms significantly affects the pullout force. As for the pullout displacement of HCNTs from the matrix, the elongation of the HCNTs was removed from the overall displacement where the elongation of HCNTs and the displacement of the loading end of HCNTs is calculated in every time step. In order to obtain the accurate pullout displacement of HCNTs, a cross-section was defined as the initial position of the loading end of HCNTs. Moreover, the pullout process of nanofillers from the matrix was captured to study the mechanism of the crack resistance of nanofillers.

### 4.1. Mechanical Properties of Composites with HCNT, Snake-like HCNT, and CNT

**Figure 3:**
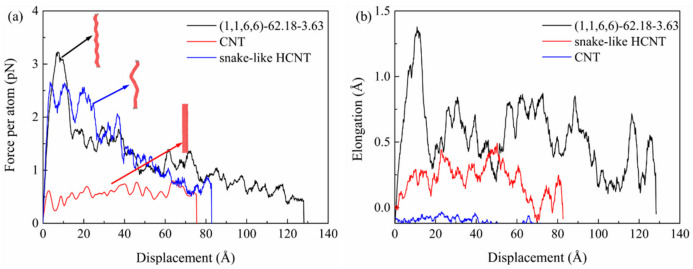
(**a**) and (**b**) The pullout force–displacement curves and elongation of HCNT–displacement curves for HCNTs (1,1,6,6)-62.18-3.63, CNTs, and snake-like HCNTs, respectively.

The composite model reinforced by the HCNT with a large pitch length, snake-like HCNT, and straight CNT was constructed to study the interfacial strength between the matrix and nanofillers. As shown in Figure 3a, the pullout force of HCNT with a large pitch length, snake-like HCNT, and straight CNT from the matrix shows the strong oscillations that took place during the pullout process. The reason for this is that the interaction area between the nanofillers and matrix reduces with the increase in pullout displacement. However, the pullout force of HCNT from the matrix is six times larger than that of CNT, which demonstrates that HCNT can provide a much better crack resistance ability than CNT due to the coarse surface of HCNT, although there are no obvious spring-like structures of HCNT. Moreover, the pullout force of the snake-like HCNT is also much larger than that of straight CNT (about five times) but smaller than that of HCNT. The results illustrate how the physical interlock between CNT and the matrix is very weak and cannot provide an effective crack resistance ability, which is also observed in experiments [47], where the CNT can be pulled out from the matrix directly.

**Figure 4:**
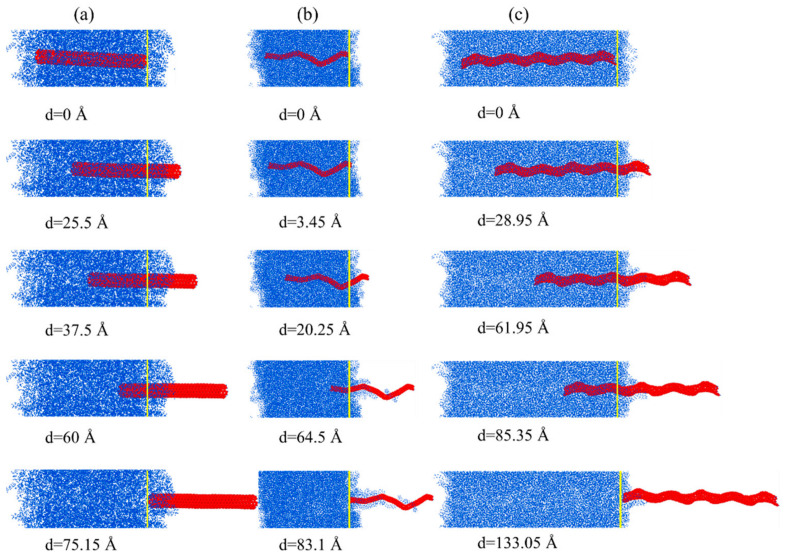
The pullout process of (**a**) straight CNTs, (**b**) snake-like HCNTs, and (**c**) HCNTs.

Moreover, the elongations of the nanofillers that were obtained during the pullout process give insight into the mechanism of the crack resistance of different reinforcements. It can be seen from Figure 3b that HCNT is obviously elongated during the pullout process. In the initial stage, the elongation of HCNT increases linearly with the increase in displacement. During this stage, one end of the HCNT is slowly pulled out from the matrix, and another end of the HCNT remains fixed in the matrix since there is strong interfacial strength between the HCNT and matrix, as shown in Figure 4c. When the loading end of the HCNT is pulled out from the matrix, there is a significant drop in the elongation and pullout force. Then, the elongation shows strong oscillation, which means that once the part of the HCNT is pulled out from the matrix, there is a recovery in the length of the HCNT, resulting in the reduction in the pullout force. Moreover, there is also an obvious elongation of snake-like HCNTs, as shown in Figure 3b, because the irregular curves of CNT segments induced by the incorporation of defects are more easily elongated. Unlike the HCNT, the elongation of snake-like HCNTs does not show a linear increase in the initial stage and reaches the maximum value at the displacement of around 50 Å, as there is a severe bending joint at the position 50 Å to the right end. As shown in Figure 4b, after the first bending joint is pulled out from the matrix at the displacement of 64.5 Å, the polymer chains are pulled out and attached with the snake-like HCNT, which means that the physical interlock induced by the bending joints is very strong. Hence, the pullout force shows a strong oscillation instead of a dramatic drop pattern before the bending joints are pulled out from the matrix. After the snake-like HCNT is completely pulled out from the matrix, it can be seen from Figure 4b that some polymer chains are attached to the surface of snake-like HCNTs. By contrast, the length of the CNT almost remains unchanged during the pullout process because there is no physical interlocking between the CNT and matrix, which makes the CNT pull out easily from the matrix [47]. A similar phenomenon is also observed in Figure 4a, where the CNT is directly pulled out from the matrix without any polymer chains attached to the surface.

### 4.2. The Effect of the Diameter of HCNTs on the Interfacial Properties

**Figure 5:**
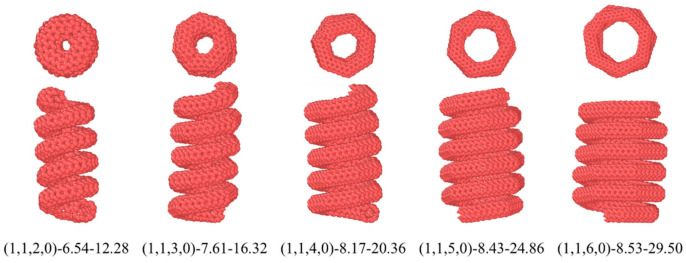
The top and front view of HCNTs with a small pitch length and different diameters where the inter-tube gap is nearly equal to the vdW interaction distance, hindering the insertion of polymer chains into the inter-tube gap.

To investigate the effect of the diameter of HCNTs on the interfacial mechanical properties, five HCNTs with a similar pitch length were constructed based on the CM3 construction method, as shown in Figure 5, where there are six coils for each HCNT. It can be seen from Figure 5 that the pitch length of all HCNTs is similar, and the inter-tube gap is nearly equal to the vdW interaction distance. Hence, the polymer chains cannot enter the inter-tube gap during the construction procedure of the molecular models of composites. Furthermore, for (1,1,2,0)-6.54-12.28, the diameter is too small to allow the filling of the polymer chains in the inner space, while the number of polymer chains filling in the inner space of other HCNTs increases with the increase in diameter. The geometrical parameters are shown in Table 1.

**Figure 6:**
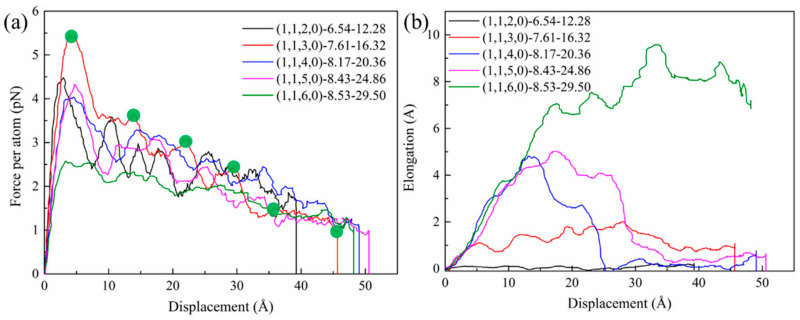
(**a**) The pullout force–displacement curves and (**b**) the elongation–displacement curves of HCNTs with different diameters during the pullout process.

The pullout force–displacement curves of HCNTs with different diameters are shown in Figure 6a. It can be observed that there are oscillations of the pullout force, and there is a linear increase in the pullout force during the initial stage for all HCNTs, where the deformation is believed to be reversible after releasing the loading. The pullout displacement before the irreversible deformation occurs is called elastic displacement, and the corresponding force is called the reversible pullout force. Moreover, the friction coefficient is calculated by fitting the displacement of 0 to reversible displacement, which is used to identify the ability to prevent the initiation of cracks. The detailed interfacial mechanical properties are shown in Table 2.

**Figure 7:**
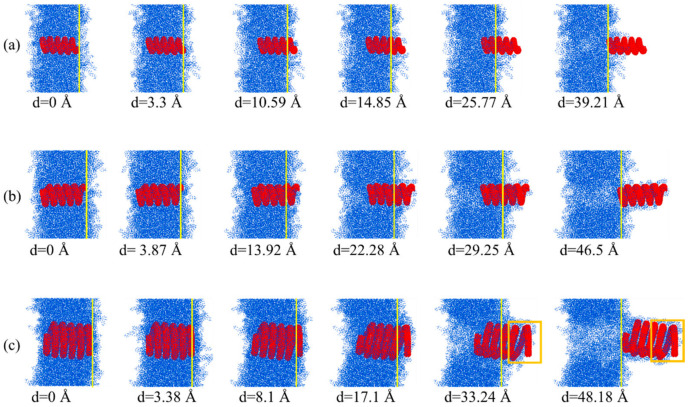
The pullout process of the HCNTs (**a**) (1,1,2,0)-6.54-12.28, (**b**) (1,1,3,0)-7.61-16.32, and (**c**) (1,1,6,0)-8.53-29.50 from the polymer matrix. There is no elongation of (1,1,2,0)-6.54-12.28 and no large number of polymer chains attached to the surface (1,1,2,0)-6.54-12.28 while (1,1,6,0)-8.53-29.50 shows large elongation and does not recover to its original length after it is completely pulled out from the matrix because there are a large number of polymer chains filled in the inter-tube space.

As shown in Figure 6a, the HCNT (1,1,3,0)-7.61-16.32 possesses the largest pullout force, and there are six stages of the pullout force during the pullout process, which is caused by the gradual pullout of HCNTs from the matrix. In the first stage, the pullout force shows a linear increase from 0 Å to about 3.86 Å, and this force is mainly controlled by the interfacial strength and the entanglement of polymer chains filled in the inner space of (1,1,3,0)-7.61-16.32 with other chains because the polymer chains can be filled in the inner space of HCNTs due to their larger diameter, as shown in Figure 7b. There is no obvious sliding deformation between the HCNT and matrix during the first stage. Meanwhile, the elongation of HCNTs shows a linear increase, as shown in Figure 6b, which means that the loading end of HCNTs begins to move towards the outside of the matrix while another end remains unmoved, as shown in Figure 7b. Then, a steep decrease in the pullout force is observed at about 3.86 Å because (1,1,3,0)-7.61-16.32 starts to slide along the pullout direction, resulting in the lessening of the interlocking of polymer chains and their association with HCNTs. The sliding of HCNTs is also indicated by the decrease in the elongation of HCNTs. With the increase in the pullout displacement, the first coil is gradually pulled out from the matrix, and the pullout force shows an oscillation in the lower range before the displacement of 13.92 Å. When the first coil in the loading end is pulled out from the matrix, the pullout force shows a steep decrease at about 13.92 Å. Along with the sequential pullout of the coils, there are six decrease patterns in the pullout force–displacement curves because there are six coils of (1,1,3,0)-7.61-16.32. It is also observed that there are oscillations in every stage instead of a significant decrease in pullout force because the physical interlock is very strong between coils, and the elongation and recovery of the length of coils happen circularly during the pullout process of every coil from the matrix.

**Figure 8:**
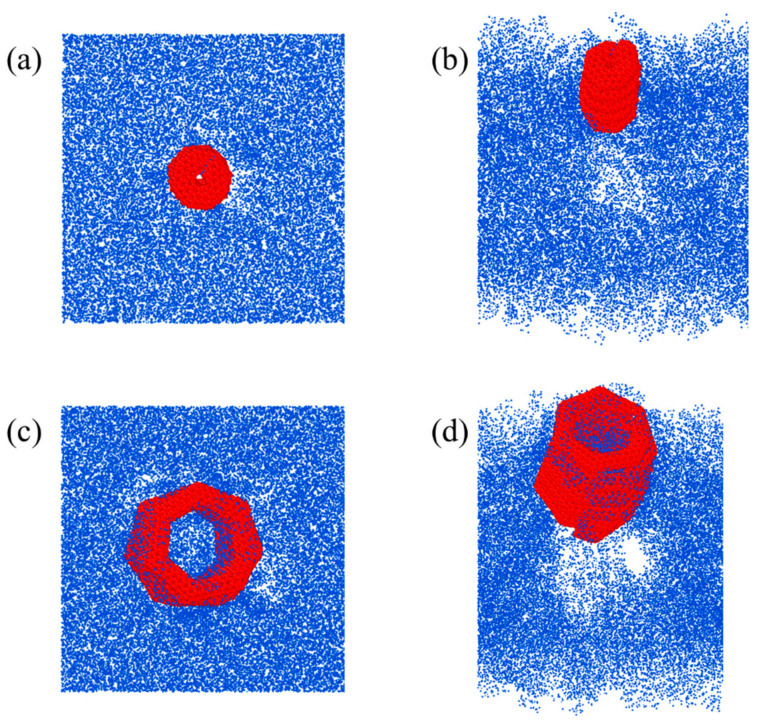
(**a**) and (**b**) The top and oblique views of composites with (1,1,2,0)-6.54-12.28; (**c**) and (**d**) The top and oblique view of composites with (1,1,6,0)-8.53-29.50 after complete pullout from the matrix. There are many polymer chains filled in the inner space of (1,1,6,0)-8.53-29.50, and a large number of polymer chains are pulled out from the matrix, resulting in large voids inside the matrix, which was not observed for (1,1,2,0)-6.54-12.28.

By contrast, the pullout force of (1,1,2,0)-6.54-12.28 showed a strong sawtooth pattern, as shown in Figure 6a. Similarly to (1,1,3,0)-7.61-16.32, the pullout force of (1,1,2,0)-6.54-12.28 showed a steep drop at the displacement of 3.29 Å after the loading end of the HCNT started to move towards the outside of the matrix, as shown in Figure 7a. Following that, the pullout force began to increase again to a second peak before the displacement of 10.39 Å. Thereafter, the first coil was completely pulled out from the matrix, inducing a steep drop in the force. The sequential pullout of the coils resulted in the oscillation of forces. It is noted that the diameter of (1,1,2,0)-6.54-12.28 was too small to allow the filling of polymer chains in the inner space illustrated in Figure 7a and Figure 8a, and strong vdW interactions between the coils hindered the separation of coils from (1,1,2,0)-6.54-12.28. Hence, (1,1,2,0)-6.54-12.28 can be seen as a straight CNT with a coarse surface, which also indicates no obvious elongation of (1,1,2,0)-6.54-12.28, as shown in Figure 6b, and the polymer chains attached on the surface of HCNTs were not observed when (1,1,2,0)-6.54-12.28 was completely pulled out from the matrix, as illustrated in Figure 8a,b.

As shown in Figure 6a, the pullout force of (1,1,6,0)-8.53-29.50 shows a different pattern to that of (1,1,3,0)-7.61-16.32 and (1,1,2,0)-6.54-12.28 where there are no obvious sawtooth patterns of pullout force–displacement curves and (1,1,6,0)-8.53-29.50 has the smallest pullout force per atom which means that (1,1,6,0)-8.53-29.50 cannot effectively hinder the initiation of crack-like (1,1,3,0)-7.61-16.32, but can hinder the crack propagation even when part of (1,1,6,0)-8.53-29.50 is pulled out from the matrix. There are three stages for the pullout force of (1,1,6,0)-8.53-29.50, as depicted in Figure 6a, where the force increases with the increase in displacement, then enters a yield phase, during which the force oscillates within a small range, and finally drops slowly to 0. In the first stage, the pullout process is similar to that of other HCNTs. When the first coil is pulled out from the matrix, there is no obvious decrease in the pullout force. The reason for this is that the larger diameter of (1,1,6,0)-8.53-29.50 enables the filling of polymer chains in the inner space of HCNTs, as shown in Figure 8c,d, and strong interfacial interactions exist between the polymer chains encapsulated within the HCNT and those in the surrounding matrix, as well as between the HCNTs themselves and the polymeric material both internally and externally. Hence, (1,1,6,0)-8.53-29.50 is consistently elongated during the pullout process, as illustrated in Figure 6b. Remarkably, as shown in Figure 7c, the space resulting from the separation of coils enables the infiltration of polymer chains that can interact with the polymers both inside and outside the HCNT, and the polymer chains in the space between the separated coils hinders the recovery of the length of (1,1,6,0)-8.53-29.50, even when it is completely pulled out from the matrix, as shown in Figure 8d. Moreover, a substantial quantity of polymer chains inside and outside HCNTs are pulled out, leading to a large void in the matrix, as shown in Figure 8d.

As shown in Table 2, the friction coefficient showed a decreasing pattern with the increase in the effective diameter of HCNTs when the diameter was larger than 16.32 Å because the large diameter meant that the lower stiffness of the HCNT itself and the increase in the number of the polymer chains filled in the inner space of HCNTs with the increase in diameter could disrupt the integrity of the matrix. A similar pattern was also observed for the maximum pullout force of HCNTs. However, the elongation of HCNTs increased with the increase in the diameters during the process, which suggests that HCNTs with large diameter can effectively hinder crack propagation once the crack forms inside the matrix.

### 4.3. The Interfacial Properties of HCNTs with Different Pitch Lengths

**Figure 9:**
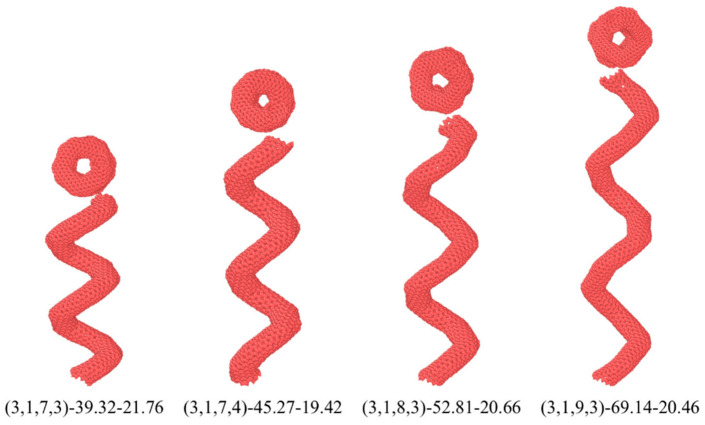
The molecular models of HCNTs with a similar diameter and different pitch lengths.

For the spring-like structure, the pitch length had a significant effect on the mechanical properties of HCNTs. Herein, in order to study the effect of the pitch length on the crack resistance ability of HCNTs in composites, four HCNTs with similar diameters and different pitch lengths were constructed, as shown in Figure 9. The detailed geometrical parameters of HCNTs are listed in Table 3, where the diameter of all HCNTs is equal to about 20 Å, and the pitch length is in the range of 39.32 Å to 69.14 Å. Herein, the pitch length starts from 39.32 Å because HCNTs are constructed based on distorted toroidal CNTs with different diameters and distortion degrees, which cannot provide the precise diameter of the macro springs. Moreover, the increase in the pitch length needs more carbon atoms, causing a very high computational cost. Hence, four representative HCNT models with two coils were constructed.

**Figure 10:**
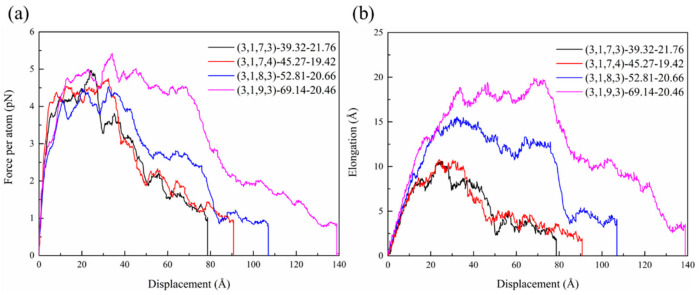
(**a**) The pullout force–displacement curves and (**b**) elongation of HCNT–displacement curves for HCNTs with different pitch lengths.

As depicted in Figure 10, the pullout force–displacement curves for HCNTs with varying pitch lengths exhibited different trends compared to HCNTs with shorter pitches, where the maximum pullout force of HCNTs with a large pitch length did not occur in the initial stage. The pullout force–displacement curves can be divided into three stages. In the first stage, the pullout force increases linearly and dramatically with the increase in displacement. Subsequently, it enters a yielding phase characterized by fluctuations in the force within a specific range. Finally, the pullout force drops to zero.

**Figure 11:**
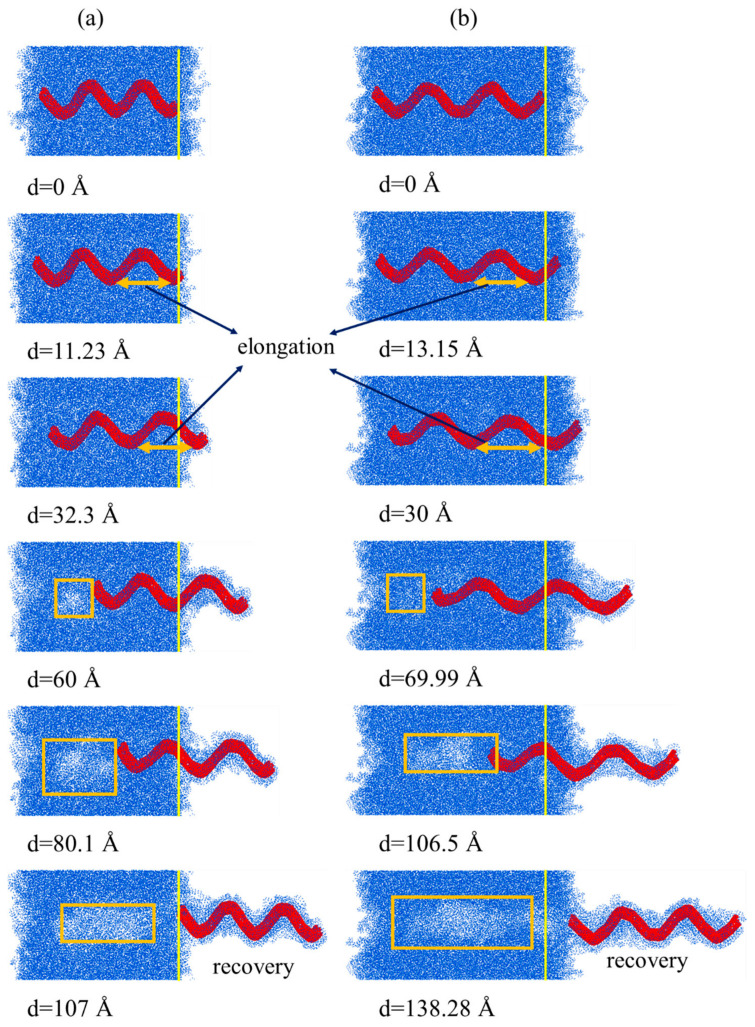
The pullout process of (**a**) (3,1,8,3)-52.81-20.66 and (**b**) (3,1,9,3)-69.14-20.46, where the polymer chains around HCNTs are pulled out from the matrix and the space between the coils are filled with polymer chains.

As shown in Figure 10, the pullout force of (3,1,9,3)-69.14-20.46 increases linearly in the range of 0 to 13.15 Å. At this stage, the HCNT is highly elongated because the polymer chains are entwined with the whole length of the HCNT, inducing the strong physical interlock between the HCNT and matrix and hindering the movement of another end of (3,1,9,3)-69.14-20.46, as shown in Figure 11b. Subsequently, a yielding stage occurs, with displacement ranging from 13.15 Å to 70 Å, indicating a transition into a phase where the pullout force fluctuates. The part near the loading end starts to be pulled out from the matrix, and the other part of the HCNT near the free end of HCNTs also starts to move. When the displacement reaches about 30 Å, the pullout force reaches the maximum value, and the elongation of (3,1,9,3)-69.14-20.46 shows a decreasing pattern after the displacement of 30 Å, where half of a coil of HCNT is pulled out from the matrix, and there is a small recovery in the length of the HCNT. The reduction in the interaction between the polymer chains around the HCNT and another polymer, as well as the interaction between the HCNT and the matrix, results in a decrease in force as the polymer chains adhering to HCNTs are also pulled out from the matrix, as illustrated in Figure 11b. When the displacement reaches about 70 Å, there is a dramatic decrease in the pullout force and elongation of HCNTs. Meanwhile, one coil of HCNT is pulled out from the matrix at a pitch length of (3,1,9,3)-69.14-20.46, which is equal to 69.14 Å. The HCNT begins to recover its original length since the interfacial interaction cannot restrain the other part of the HCNT, and the large number of polymer chains attached to the HCNT are pulled out from the matrix, as illustrated in Figure 11b.

Actually, when the whole HCNT begins to move towards the outside of the matrix at the displacement of 13.15 Å to 70 Å, the polymer chains between the coils of HCNT also start to separate from other polymer chains, which causes voids inside the matrix, as shown in Figure 11b. By contrast, the pullout of (3,1,8,3)-52.81-20.66 from the matrix only causes smaller voids, which means that the interfacial physical lock of HCNTs with a large pitch length is stronger, as shown in Figure 11a. When one coil of the HCNT is completely pulled out from the matrix, the length of the HCNT begins to recover, and the polymer chains located between the coils of the HCNT are totally separated from the other polymer chains. Thereafter, the polymer chains filling in space between the coils and HCNT can be seen as an integrated structure since there is no sliding or relative movement between them. Hence, the physical interlock between the HCNT and matrix changes the relatively weak interaction between the polymer chains in the coils and other polymer chains in the matrix, resulting in a steep decrease in the force after the displacement reaches over 70 Å. When (3,1,9,3)-69.14-20.46 is completely pulled out from the matrix, a relatively smooth plane is observed on the surface of the HCNT where the space between coils is fully filled by polymer chains, as shown in Figure 11b. Meanwhile, a large void forms inside the matrix.

In Figure 10, the pullout force of (3,1,7,3)-39.32-21.76, (3,1,7,4)-45.27-19.42, and (3,1,8,3)-52.81-20.66 also show a similar pattern to that of (3,1,9,3)-69.14-20.46. Distinct differences are observed during the yielding stage, where the pullout force of (3,1,9,3)-69.14-20.46 maintains a high value within the large displacement range while the force of other HCNTs shows a rapidly decreasing pattern. Furthermore, the reversible displacement and force of HCNTs increase with the increase in pitch length, as shown in Table 4, which means that the composites reinforced by HCNTs with a larger pitch length possess better crack resistance. On the other hand, the friction coefficient shows an opposite pattern, which suggests that the HCNTs with a smaller pitch length can hinder crack initiation in a small displacement range.

## 5. Conclusions

The interfacial mechanical properties of nanocomposites reinforced by CNTs and HCNTs with different diameters and helix angles were investigated through the pullout test. The interfacial characteristics exhibited significant variations among the nanocomposites, incorporating nanofillers with different geometries, which can significantly influence their overall performance.

(a) The shear resistance of HCNTs is much better than that of straight CNTs during the pullout process, where the maximum pullout force per atom of the CNT is only 0.8 pN which is four times smaller than that of HCNTs (up to 3.23 pN). However, (1,1,2,0)-6.54-12.28 with both a small diameter and helix angle showed very poor interfacial mechanical properties, which are similar to CNTs.
(b) As for the nanocomposites reinforced by HCNTs with different diameters, the friction coefficient showed a decreasing pattern with the increase in the diameter of HCNTs. And (1,1,3,0)-7.61-16.32 possessed the largest maximum pullout force (5.45 pN) while (1,1,6,0)-8.43-29.50 had the smallest maximum pullout force (2.57 pN). Furthermore, (1,1,2,0)-6.54-12.28 with the smallest diameter and inter-tube gap showed a similar pattern with straight CNTs where the interlocking strength was very weak and could be easily pulled out from the matrix.
(c) Among the nanocomposites reinforced by HCNTs with different pitch lengths, (3,1,8,3)-52.81-20.66 with a pitch length of 52.81 Å had the ability to prevent the initiation and the growth of cracks since it simultaneously possessed a large friction coefficient, reversible force, and maximum force.
(d) During the pullout process, there was an obvious elongation of HCNTs, causing the high value and strong oscillation of the pullout force, indicating the efficient load transfer ability from the matrix to nanofillers. The elongation of HCNTs depends both on the helix angle and the stiffness of HCNTs. On the other hand, the length of straight CNTs remains unchanged during the process, which can also be observed for snake-like HCNTs and (1,1,2,0)-6.54-12.28 with both a small diameter and pitch length.
(e) After the nanofillers are pulled out from the matrix, HCNTs can carry out the most polymer chains, and an even bigger void is generated inside the polymer matrix because of the biggest diameter being used while the CNT cannot capture polymer chains efficiently. This indicates that the shear fracture of nanocomposites for HCNTs always occurs inside the matrix, while fracture only occurs on the interface for nanocomposites reinforced by CNTs with a large helix angle.

The findings in this research reveal that HCNTs with appropriate geometrical properties can provide efficient shear resistance in the nanocomposites while snake-like HCNTs and (1,1,2,0)-6.54-12.28 with both a small diameter and pitch length, showing similar interfacial mechanical properties with straight CNTs, which can provide guidance to fabricate nanocomposites with desirable mechanical properties.

## Figures and Tables

**Table 1:** The geometrical properties of HCNTs with different diameters.

HCNTs	Number of Atoms	Tube Radius *d* (Å)	Effective Diameter *D* (Å)	Pitch Length *p* (Å)	Helix Angle α (°)
(1,1,2,0)-6.54-12.28	1164	2.7	12.28	6.54	4.84
(1,1,3,0)-7.61-16.32	1744	2.7	16.32	7.61	4.24
(1,1,4,0)-8.17-20.36	2320	2.7	20.36	8.17	3.65
(1,1,5,0)-8.43-24.86	2896	2.7	24.86	8.43	3.09
(1,1,6,0)-8.53-29.50	3472	2.7	29.50	8.53	2.63

**Table 2:** The mechanical properties of nanocomposites reinforced by HCNTs with different diameters.

HCNTs	Reversible Displacement (Å)	Reversible Force (pN)	Friction Coefficient (pN/Å)	Maximum Pullout Force (pN)	Maximum Elongation (Å)
(1,1,2,0)-6.54-12.28	3.29	4.48	1.36	4.48	0.23
(1,1,3,0)-7.61-16.32	3.86	5.45	1.41	5.45	2.01
(1,1,4,0)-8.17-20.36	3.96	4.04	1.02	4.04	4.81
(1,1,5,0)-8.43-24.86	4.78	4.33	0.91	4.33	5.02
(1,1,6,0)-8.53-29.50	3.38	2.57	0.76	2.57	9.89

**Table 3:** The geometrical properties of HCNTs with different pitch lengths.

HCNTs	Number of Atoms	Tube Radius *d* (Å)	Effective Diameter *D* (Å)	Pitch Length *p* (Å)	Helix Angle α (°)
(3,1,7,3)-39.32-21.76	1754	4.2	21.76	39.32	16.05
(3,1,7,4)-45.27-19.42	1754	4.2	19.42	45.27	20.35
(3,1,8,3)-52.81-20.66	2042	4.2	20.66	52.81	22.13
(3,1,9,3)-69.14-20.46	2330	4.2	20.46	69.14	28.27

**Table 4:** The mechanical properties of composites reinforced by HCNTs with different pitch lengths.

HCNTs	Reversible Displacement (Å)	Reversible Force (pN)	Friction Coefficient (pN/Å)	Maximum Pullout Force (pN)	Maximum Elongation (Å)
(3,1,7,3)-39.32-21.76	5.83	3.83	0.66	4.97	10.95
(3,1,7,4)-45.27-19.42	8.06	4.11	0.51	4.75	11.08
(3,1,8,3)-52.81-20.66	11.23	4.10	0.37	4.52	25.63
(3,1,9,3)-69.14-20.46	13.15	4.76	0.36	5.42	19.90

## Data Availability

Data is contained within the article.

## References

[1] Ding P., Wu J., Zhang J., Shao J., Tang W., Hou G., Zhang L., Chen X. (2020). Role of geometric shapes on the load transfer in graphene-PMMA nanocomposites. Comput. Mater. Sci..

[2] Kumar A., Sharma K., Dixit A.R. (2019). A review on the mechanical and thermal properties of graphene and graphene-based polymer nanocomposites: Understanding of modelling and MD simulation. Mol. Simul..

[3] Izadi R., Ghavanloo E., Nayebi A. (2019). Elastic properties of polymer composites reinforced with C60 fullerene and carbon onion: Molecular dynamics simulation. Phys. B Condens. Matter.

[4] Yang S., Yu S., Cho M. (2013). Influence of Thrower–Stone–Wales defects on the interfacial properties of carbon nanotube/polypropylene composites by a molecular dynamics approach. Carbon.

[5] Nie F., Jian W., Lau D. (2021). An atomistic study on the thermomechanical properties of graphene and functionalized graphene sheets modified asphalt. Carbon.

[6] Li Y., Wang S., Wang Q., Xing M. (2018). A comparison study on mechanical properties of polymer composites reinforced by carbon nanotubes and graphene sheet. Compos. Part B Eng..

[7] van de Werken N., Tekinalp H., Khanbolouki P., Ozcan S., Williams A., Tehrani M. (2020). Additively manufactured carbon fiber-reinforced composites: State of the art and perspective. Addit. Manuf..

[8] Shen S., Yang L., Wang C., Wei L. (2020). Effect of CNT orientation on the mechanical property and fracture mechanism of vertically aligned carbon nanotube/carbon composites. Ceram. Int..

[9] Zhang W., Deng X., Sui G., Yang X. (2019). Improving interfacial and mechanical properties of carbon nanotube-sized carbon fiber/epoxy composites. Carbon.

[10] Abidin M.S.Z., Herceg T., Greenhalgh E.S., Shaffer M., Bismarck A. (2019). Enhanced fracture toughness of hierarchical carbon nanotube reinforced carbon fibre epoxy composites with engineered matrix microstructure. Compos. Sci. Technol..

[11] Zhang L.W., Ji W.M., Liew K.M. (2018). Mechanical properties of diamond nanothread reinforced polymer composites. Carbon.

[12] Rouhi S., Alizadeh Y., Ansari R. (2014). On the interfacial characteristics of polyethylene/single-walled carbon nanotubes using molecular dynamics simulations. Appl. Surf. Sci..

[13] Duan K., Li L., Wang F., Liu S., Hu Y., Wang X. (2021). New insights into interface interactions of CNT-reinforced epoxy nanocomposites. Compos. Sci. Technol..

[14] Wang J.F., Li P.H., Tian X.B., Shi S.Q., Tam L.-H. (2023). Molecular investigation on temperature-dependent mechanical properties of PMMA/CNT nanocomposite. Eng. Fract. Mech..

[15] Wang J.F., Shi S.Q., Liu Y.Z., Yang J.P., Tam L.-H. (2022). Multiscale simulation of temperature- and pressure-dependent nonlinear dynamics of PMMA/CNT composite plates. Nonlinear Dyn..

[16] Tang L.-C., Zhang H., Wu X.-P., Zhang Z. (2011). A novel failure analysis of multi-walled carbon nanotubes in epoxy matrix. Polymer.

[17] Quan D., Urdániz J.L., Ivanković A. (2018). Enhancing mode-I and mode-II fracture toughness of epoxy and carbon fibre reinforced epoxy composites using multi-walled carbon nanotubes. Mater. Des..

[18] Zheng Q.B., Xue Q.Z., Yan K.O., Hao L.Z., Li Q., Gao X.L. (2007). Investigation of molecular interactions between SWNT and polyethylene/polypropylene/polystyrene/polyaniline molecules. J. Phys. Chem. C.

[19] Liu F., Hu N., Ning H., Liu Y., Li Y., Wu L. (2015). Molecular dynamics simulation on interfacial mechanical properties of polymer nanocomposites with wrinkled graphene. Comput. Mater. Sci..

[20] Awasthi A.P., Lagoudas D.C., Hammerand D.C. (2009). Modeling of graphene–polymer interfacial mechanical behavior using molecular dynamics. Model. Simul. Mater. Sci. Eng..

[21] Li Y., Seidel G.D. (2014). Multiscale modeling of the effects of nanoscale load transfer on the effective elastic properties of unfunctionalized carbon nanotube–polyethylene nanocomposites. Model. Simul. Mater. Sci. Eng..

[22] Mahboob M., Islam M.Z. (2013). Molecular dynamics simulations of defective CNT-polyethylene composite systems. Comput. Mater. Sci..

[23] Islam M.Z., Mahboob M., Lowe R.L. (2016). Mechanical properties of defective carbon nanotube/polyethylene nanocomposites: A molecular dynamics simulation study. Polym. Compos..

[24] Peng X., Meguid S.A. (2017). Molecular simulations of the influence of defects and functionalization on the shear strength of carbon nanotube-epoxy polymer interfaces. Comput. Mater. Sci..

[25] Kai M.F., Zhang L.W., Liew K.M. (2020). Carbon nanotube-geopolymer nanocomposites: A molecular dynamics study of the influence of interfacial chemical bonding upon the structural and mechanical properties. Carbon.

[26] Kai M.F., Zhang L.W., Liew K.M. (2019). Graphene and graphene oxide in calcium silicate hydrates: Chemical reactions, mechanical behavior and interfacial sliding. Carbon.

[27] Jian W., Lau D. (2020). Understanding the effect of functionalization in CNT-epoxy nanocomposite from molecular level. Compos. Sci. Technol..

[28] Sharma K., Kaushalyayan K.S., Shukla M. (2015). Pull-out simulations of interfacial properties of amine functionalized multi-walled carbon nanotube epoxy composites. Comput. Mater. Sci..

[29] Li Y., Seidel G.D. (2015). Multiscale modeling of functionalized interface effects on the effective elastic material properties of CNT–polyethylene nanocomposites. Comput. Mater. Sci..

[30] Karimi M.R., Abrinia K., Hamdia K.M., Hashemianzadeh S.M., Baniassadi M. (2022). Effects of functional group type and coverage on the interfacial strength and load transfer of graphene-polyethylene nanocomposites: A molecular dynamics simulation. Appl. Phys. A.

[31] Yuan Z., Lu Z., Chen M., Yang Z., Xie F. (2015). Interfacial properties of carboxylic acid functionalized CNT/polyethylene composites: A molecular dynamics simulation study. Appl. Surf. Sci..

[32] Lau K.-T., Lu M., Liao K. (2006). Improved mechanical properties of coiled carbon nanotubes reinforced epoxy nanocomposites. Compos. Part A Appl. Sci. Manuf..

[33] Yoshimura K., Nakano K., Miyake T., Hishikawa Y., Motojima S. (2006). Effectiveness of carbon microcoils as a reinforcing material for a polymer matrix. Carbon.

[34] Li X., Lau K., Yin Y. (2008). Mechanical properties of epoxy-based composites using coiled carbon nanotubes. Compos. Sci. Technol..

[35] Sharifian A., Baghani M., Odegard G.M., Wu J., van Duin A.C.T., Baniassadi M. (2019). How to characterize interfacial load transfer in spiral carbon-based nanostructure-reinforced nanocomposites: Is this a geometry-dependent process?. Phys. Chem. Chem. Phys..

[36] Vijayan R., Ghazinezami A., Taklimi S.R., Khan M.Y., Askari D. (2019). The geometrical advantages of helical carbon nanotubes for high-performance multifunctional polymeric nanocomposites. Compos. Part B Eng..

[37] Wang Y., Mei Y., Wang Q., Wei W., Huang F., Li Y., Li J., Zhou Z. (2019). Improved fracture toughness and ductility of PLA composites by incorporating a small amount of surface-modified helical carbon nanotubes. Compos. Part B Eng..

[38] Kadhim N., Mei Y., Wang Y., Li Y., Meng F., Jiang M., Zhou Z. (2018). Remarkable Improvement in the Mechanical Properties of Epoxy Composites Achieved by a Small Amount of Modified Helical Carbon Nanotubes. Polymers.

[39] Sharifian A., Baghani M., Wu J., Odegard G.M., Baniassadi M. (2019). Insight into Geometry-Controlled Mechanical Properties of Spiral Carbon-Based Nanostructures. J. Phys. Chem. C.

[40] Sarapat P., Hill J., Baowan D. (2019). A review of geometry, construction and modelling for carbon nanotori. Appl. Sci..

[41] Wu J., Zhao H., Liu J., Zhang Z., Ning F., Liu Y. (2018). Nanotube-chirality-controlled tensile characteristics in coiled carbon metastructures. Carbon.

[42] Liu X., Yang Q.S., Liew K.M., He X.Q. (2017). Superstretchability and stability of helical structures of carbon nanotube/polymer composite fibers: Coarse-grained molecular dynamics modeling and simulation. Carbon.

[43] Chang X., Xu Q., Lv J., Xu L., Zhu Z., Liu S., Liu X., Qin J. (2021). Bioinspired 3D helical fibers toughened thermosetting composites. Compos. Part B Eng..

[44] Chuang C., Fan Y.-C., Jin B.-Y. (2009). Generalized classification scheme of toroidal and helical carbon nanotubes. J. Chem. Inf. Model..

[45] Haghighi S., Ansari R., Ajori S. (2019). Influence of polyethylene cross-linked functionalization on the interfacial properties of carbon nanotube-reinforced polymer nanocomposites: A molecular dynamics study. J. Mol. Model..

[46] Nikkhah S.J., Moghbeli M.R., Hashemianzadeh S.M. (2016). Dynamic study of deformation and adhesion of an amorphous polyethylene/graphene interface: A simulation study. Macromol. Theory Simul..

[47] Qin R., Zhou A., Yu Z., Wang Q., Lau D. (2021). Role of carbon nanotube in reinforcing cementitious materials: An experimental and coarse-grained molecular dynamics study. Cem. Concr. Res..

